# Exploring Male-Specific Synaptic Plasticity in Major Depressive Disorder: A Single-Nucleus Transcriptomic Analysis Using Bioinformatics Methods

**DOI:** 10.3390/ijms26073135

**Published:** 2025-03-28

**Authors:** Ji Chen, Xiumei Zhu, Fan Yang, Yanan Liu, Huajie Ba, Ping Huang, Hongyan Wang, Yingnan Bian, Chengtao Li, Suhua Zhang

**Affiliations:** 1 Institute of Forensic Science, Fudan University, Shanghai 200032, China; chenji@fudan.edu.cn (J.C.); xiumeizhu1128@gmail.com (X.Z.); huangping@fudan.edu.cn (P.H.); 2 School of Forensic Medicine, China Medical University, Shenyang 110122, China; 3 Key Laboratory of Forensic Evidence and Science Technology, Institute of Forensic Science, Ministry of Public Security, Shanghai 200042, China; fan_yang_27@163.com (F.Y.); dyndai@sina.cn (Y.L.); 4 DNA Laboratory, Public Security Bureau of Changzhou, Changzhou 213022, China; bahuajie@163.com; 5 Institute of Metabolism and Integrative Biology, Institute of Reproduction and Development, Institutes of Biomedical Sciences, Fudan University, Shanghai 200032, China; wanghy@fudan.edu.cn; 6 Enlight Medical Technologies, Pudong New Area, Shanghai 201318, China; yingnan_bian@enlight-medical.com

**Keywords:** major depressive disorder, synaptic plasticity, single-nucleus RNA sequencing, machine learning, hub gene

## Abstract

Major depressive disorder (MDD) is a complex psychiatric illness, with synaptic plasticity playing a key role in its pathology. Our study aims to investigate the molecular basis of MDD by analyzing synaptic plasticity-related gene expression at the single-cell level. Utilizing a published snRNA-seq dataset (GSE144136), we identified Excitatory.neurons_1 as the cell cluster most associated with MDD and synaptic plasticity through cell clustering, gene set enrichment analysis (GSEA), and pseudotime analysis. Integrating the bulk RNA-seq data (GSE38206), we identified CASKIN1 and CSTB as hub genes via differential expression analysis and machine learning methods. Further exploration of the relevant mechanisms was performed via cell–cell communication and ligand-receptor interaction analysis, functional enrichment analysis, and the construction of molecular regulatory networks, highlighting miR-21-5p as a key biomarker. We propose that elevated miR-21-5p in MDD downregulates CASKIN1 in Excitatory.neurons_1 cells, resulting in decreased neural connectivity and altered synaptic plasticity. As our analyzed snRNA-seq dataset consists solely of male samples, these findings may be male-specific. Our findings shed light on potential mechanisms underlying synaptic plasticity in MDD, offering novel insights into the disorder’s cellular and molecular dynamics.

## 1. Introduction

Major depressive disorder (MDD) is a global public health concern, affecting over 300 million people across all age groups. It is a leading cause of disability worldwide, contributing substantially to the global disease burden [1]. Severe cases of MDD can result in suicide, which claims approximately 800,000 lives annually, making it the second leading cause of death among individuals aged 15–29 [2]. Despite the high prevalence and severe impact of MDD, current diagnostic and treatment methods have significant limitations. Many patients do not respond adequately to existing treatments, and diagnoses often rely on subjective assessments rather than objective biomarkers [3].

MDD is a heterogeneous and multifactorial disorder, and despite extensive research, no single mechanism fully explains all of its aspects [4]. Current hypotheses about MDD’s pathogenesis include the decline of the monoamine transmitter system [5], enhancement of the hypothalamic–pituitary–adrenal (HPA) axis [6], inflammatory activation [7], decreased neuroplasticity and neurogenesis, changes in brain structure and function [8], and the involvement of genes, environmental factors, and epigenetics [9]. Among these, the synaptic plasticity hypothesis is particularly noteworthy, as it may link various changes observed in MDD [10].

Synaptic plasticity refers to the enduring structural and functional changes in the strength and efficacy of synaptic connections between neurons in response to activity or environmental changes [11,12]. It underpins the brain’s ability to perceive, assess, and store complex information, as well as to make adaptive responses to stimuli [7,13]. Increasing evidence suggests that synaptic plasticity is impaired in MDD [10,14,15]. Studies have shown the reduced synaptic density and decreased expression of synaptic proteins in the dorsolateral prefrontal cortex and hippocampus of MDD patients [16,17], indicating potential impairment in synaptic plasticity. Key biomarkers for synaptic plasticity and neuronal growth, such as brain-derived neurotrophic factor (BDNF) and mammalian target of rapamycin (mTOR), exhibit significant alterations in MDD [4,10]. Additionally, antidepressant drugs may enhance synaptic plasticity. For example, selective serotonin-reuptake inhibitors (SSRIs) and tricyclic antidepressants (TCAs) improve synaptic plasticity [18,19], and the ketamine treatment has been shown to restore synaptic plasticity and ameliorate MDD-related behaviors by modulating TIAM1 [20]. However, the precise molecular mechanisms underlying changes in synaptic plasticity in MDD, particularly the differences and dynamic changes among various neuronal cells, remain unclear.

Gene expression patterns in the brain are cell-type specific, with complex interactions between various types of cells [21]. Recently developed single-cell RNA sequencing (scRNA-seq) [22] and single-nucleus RNA sequencing (snRNA-seq) [23] provide solutions to the challenge of determining whether subtle molecular differences observed in tissue homogenates are due to disease states or variations in cell composition between samples. These techniques allow us to study gene expression patterns in the human brain at the single-cell or single-nucleus level, offering new insights into the mechanisms of synaptic plasticity in MDD.

This study aimed to explore the potential molecular characteristics of MDD by focusing on the expression of genes related to synaptic plasticity at the single-cell level. The workflow of this study is shown in Figure S1. We employed a range of bioinformatics methods, including snRNA-seq data analysis, synaptic plasticity-related gene scoring (Table S1), pseudotime trajectory analysis, cell communication analysis, gene ontology (GO), and Kyoto Encyclopedia of genes and genomes (KEGG) pathway enrichment analysis, gene set enrichment analysis (GSEA), and machine learning-based hub gene identification. These methodologies allowed us to dissect the molecular mechanisms of MDD from multiple dimensions. The primary goal of this study was to identify key genes and their regulatory networks associated with MDD, thereby providing new insights for future diagnostic and therapeutic strategies.

## 2. Results

### 2.1. Identification of 21 Cell Clusters from Single-Nucleus RNA-Seq Data

**Figure 1:**
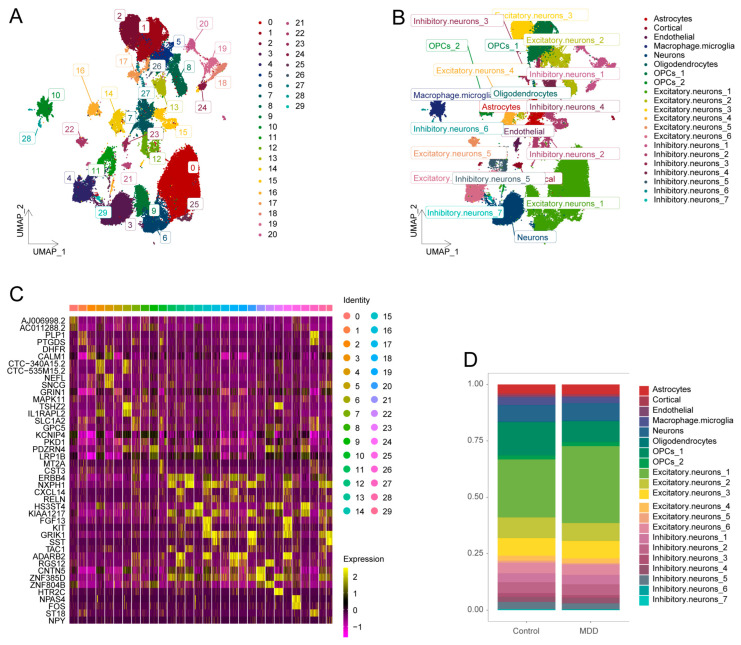
Identification of cell clusters. (**A**) The UMAP plot shows unannotated clusters in the MDD and control groups, illustrating the distribution of nuclei across the different clusters. (**B**) The UMAP plot of annotated cell clusters in both the MDD and control groups. (**C**) A heatmap depicting the expression of genes specifically associated with each unannotated cluster, highlighting the distinct gene expression profiles that differentiate these clusters. (**D**) Bar plots representing the proportions of each identified cell cluster in the MDD and control groups, demonstrating the relative abundance of different cell clusters between the two groups.

We utilized the single-nucleus RNA-seq dataset GSE144136 to analyze the cell clusters in both MDD and control groups. From a total of 78,886 nuclei, 30 distinct clusters were identified (Figure 1A). Through gene expression profiling, we annotated 21 distinct cell clusters across nine cell types (Figure 1B) using cell-specific biomarkers (Table S2). The expression of two characteristic genes in each unannotated cluster is illustrated in Figure 1C. The proportions of different cell clusters in the MDD and control groups are depicted in Figure 1D. Specific genes for each cell cluster are visualized in dot plots (Figure S2).

### 2.2. Identification of Synaptic Plasticity Activity in Different Cell Subsets

**Figure 2:**
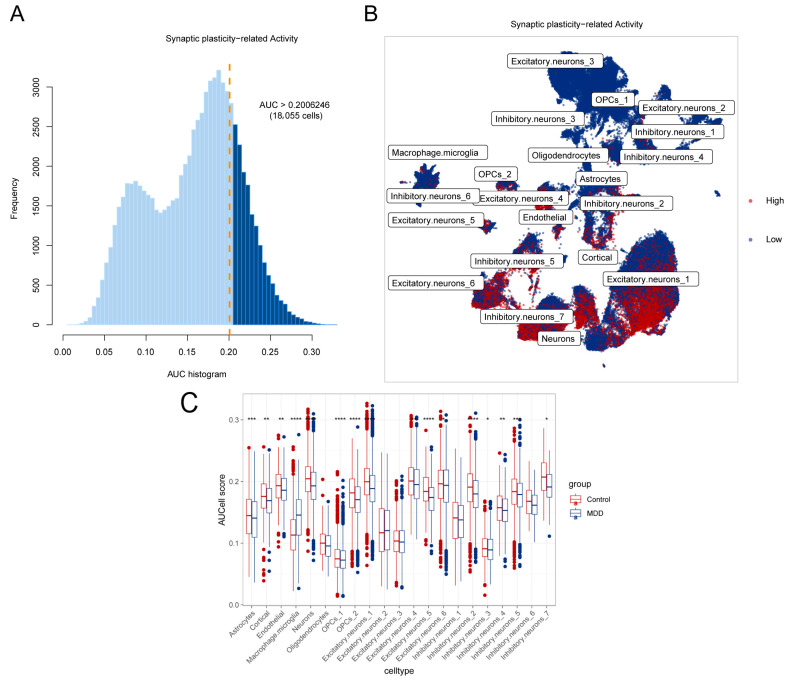
Identification of synaptic plasticity activity. (**A**) The plot illustrates the AUC threshold used to differentiate cells with high synaptic plasticity activity from those with low activity, based on the expression of synaptic plasticity-related genes. (**B**) The UMAP diagram displays the distribution of cell subsets with high and low synaptic plasticity activity, visualizing how these subsets are spatially organized and differentiated within the overall cell population. (**C**) The AUCell scores of synaptic plasticity activity in different cell clusters of the control and the MDD groups. (**** *p* < 0.0001, *** *p* < 0.001, ** *p* < 0.01, * *p* < 0.05).

To identify activated cell subsets, we analyzed synaptic plasticity-related gene expression at the single-nucleus level. Cells with an AUC value above 0.2006246 were considered to have high synaptic plasticity-related gene activity, while those below this threshold were considered to have low activity. We identified 18,055 cells with high synaptic plasticity activity (Figure 2A). And the UMAP plot illustrates cell subsets with varying synaptic plasticity activities (Figure 2B).

The AUCell scores were analyzed to compare the synaptic plasticity activity in different cell clusters. The results show that MDD led to a significant reduction in synaptic plasticity activity across a large number of cell types. Among them, Excitatory.neurons_1 and Neurons exhibited the highest activity scores, with the most significant differences observed between groups (*p* < 0.0001) (Figure 2C).

### 2.3. Pseudotime Trajectory Analysis of Cells with High Synaptic Plasticity Activity

**Figure 3:**
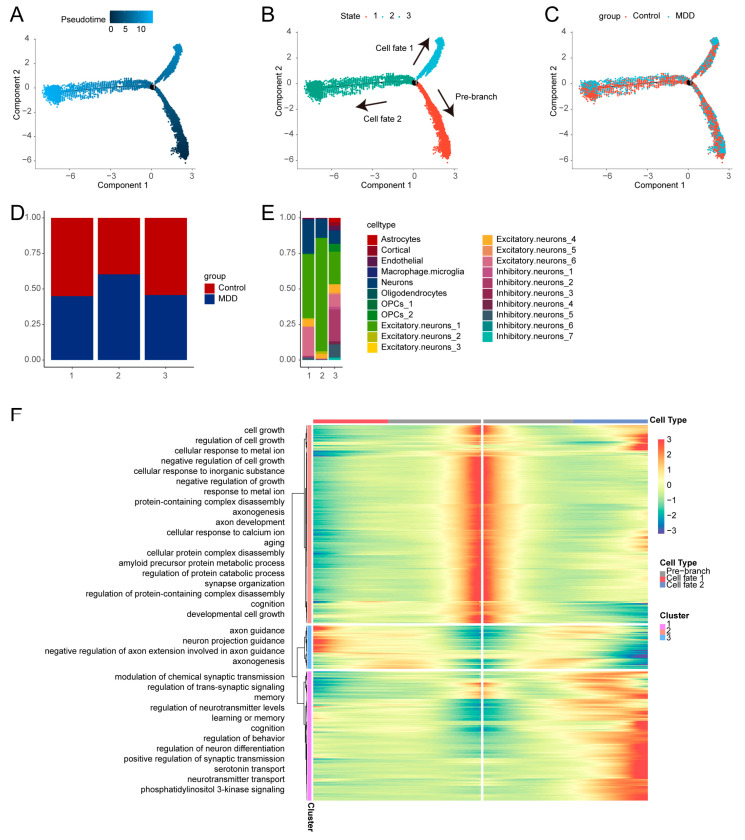
Pseudotime trajectory analysis. (**A**) The pseudotime trajectory analysis is represented with a color gradient transitioning from dark blue to light blue, indicating the progression of cellular states over pseudotime. (**B**) The pseudotime trajectory plot shows the three distinct transcriptional states identified, each represented by a separate branch in the trajectory. (**C**) The distribution of cells from the MDD and control groups is mapped along the pseudotime trajectory, highlighting differences in cell state progression between the groups. (**D**) Bar plots display the proportion of cells from the MDD and control groups within each of the three pseudotime states. (**E**) Bar plots show the distribution of different cell clusters within the three pseudotime states. (**F**) The heatmap presents the DEGs across the three pseudotime states and their associated GO pathway clusters, indicating key biological processes that are enriched in each state.

We constructed a pseudotime trajectory of cells with high synaptic plasticity activity to identify critical gene expression programs driving MDD progression. These cells formed three distinct branches, each representing a different transcriptional state (Figure 3A–C). The cells in the pre-branch state were considered initial cells, further differentiating into cells in Cell fate 1 and Cell fate 2 states. The proportions of MDD and control cells in different states are shown in Figure 3D and cell clusters in different states are shown in Figure 3E. There was a significant increase in the proportion of cells in State 2 in the MDD group, consistent with the increased proportion of Excitatory.neurons_1 cells in State 2.

We further investigated the genes defining the cellular branches associated with MDD. Genes highly expressed in the pre-branch state were enriched in GO biological processes such as “cell growth”, “cellular response to metal ion”, and “negative regulation of cell growth”. Genes in Cell fate 1 were enriched in “axon guidance”, “neuron projection guidance”, and “negative regulation of axon extension involved in axon guidance”. In Cell fate 2, genes were enriched in “modulation of chemical synaptic transmission”, “regulation of trans-synaptic signaling”, and “memory” (Figure 3F).

### 2.4. Cell–Cell Communication and Ligand-Receptor Interaction Analysis

We explored the cell–cell interaction network between the MDD and control groups to reveal changes in cell crosstalk. The MDD group showed a reduction in the total number and intensity of interactions compared to the control group (Figure S3A). Most interactions in the MDD group were decreased and weakened (Figure S3B,C). Overall signal patterns between the two groups are shown in Figure S3D, with outgoing and incoming signal patterns detailed in Figures S4 and S5, respectively.

Focused on Excitatory.neurons_1 cells, we analyzed ligand-receptor pairs regulating communication between Excitatory.neurons_1 cells and other cells. The NRG and CNTN signaling pathways were prominent in MDD. In the MDD group, the NRG3 ligand from Excitatory.neurons_1 cells bound to receptors on OPCs_1 and Excitatory.neurons_4 cells, while CNTN1 from Excitatory.neurons_1 cells bound to receptors on Macrophage.microglia cells. NRG3 ligands also bound to receptors on Inhibitory.neurons_3 cells, but this interaction was reduced in the MDD group (Figures S6 and S7).

Further analysis of the NRG signaling pathway revealed that Excitatory.neurons_1 cells in the MDD group communicated with multiple cell types through NRG1-ERBB4, NRG2-ERBB4, and NRG3-ERBB4 receptors (Figure S8A). The CNTN signaling pathway showed interactions through CNTN1 and NFASC ligands with NRCAM and CNTN1_CNTNAP1 receptors (Figure S8B). The expression levels of receptors and ligands in the NRG signaling pathway in different cell types of the MDD group are shown in Figure S8C, with increased expression of the CNTNAP2 receptor in Macrophage.microglia cells (Figure S8D).

### 2.5. Analysis of DEGs Associated with Synaptic Plasticity in MDD

We identified 1766 DEGs between Excitatory.neurons_1 cells and other cell clusters in MDD (Table S3). The top 10 up-regulated genes in Excitatory.neurons_1 cells (AC011288.2, KCNIP4, IQCJ-SCHIP1, AJ006998.2, RP11-30J20.1, CBLN2, HS6ST3, LDB2, Lingo-2, PTPRK) are displayed in a heat map (Figure S9A).

A comparison of MDD and control samples in GSE38206 revealed a total of 2262 DEGs. The top five up-regulated genes (LOC391334, FTH1, NPTN, HNRNPF, API5) and down-regulated genes (EML4, A_33_P3281563, RN7SK, SCARNA5, HIST1H4E) in the MDD samples are shown in a heat map (Figure S9B, Table S4). We identified 102 overlapping genes between the two DEG sets (Figure S9C, Table S5).

To study the biological functions related to the overlapping genes, we performed enrichment analyses of GO terms and KEGG pathways (Tables S6 and S7). The GO results showed that these genes were enriched in BP pathways such as axon extension, neuron projection extension, and the regulation of axon extension; CC pathways such as neuron-to-neuron synapse, postsynaptic density, and asymmetric synapse; and MF pathways such as transmembrane transporter binding and actin binding (Figure S9D). The enriched KEGG pathways include arrhythmogenic right ventricular cardiomyopathy, hypertrophic cardiomyopathy, and dilated cardiomyopathy (Figure S9E).

### 2.6. Identification of Two Hub Genes Using Multiple Machine Learning Methods

**Figure 4:**
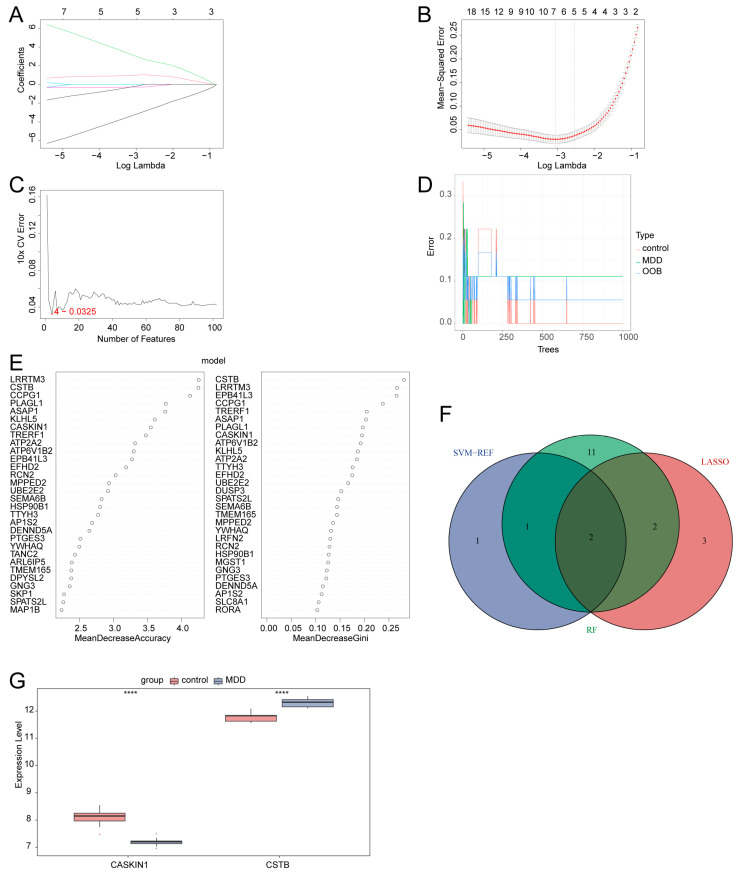
Identification of hub genes with multiple machine learning methods. (**A**) LASSO coefficient profiles of candidate genes, showing the coefficients of each gene as the tuning parameter log Lambda changes. (**B**) Cross-validation plot used to select the optimal tuning parameter log Lambda in LASSO analysis, indicating the point where the model achieves the best performance. (**C**) Identification of four genes by SVM-RFE analysis, with an accuracy of 0.9675, demonstrating the effectiveness of this method in selecting key genes. (**D**) The error rate of the RF model compared to the number of classification trees, showing how the model’s performance changes as more trees are included. (**E**) The top 30 synaptic plasticity-related DEGs ranked by importance using the RF algorithm, highlighting the most significant genes in the dataset. (**F**) Venn diagram showing the intersection of two hub genes identified through LASSO, SVM-RFE, and RF methods, indicating the consensus genes across all three approaches. (**G**) Box plot comparing the expression levels of the hub genes between the MDD group and the control group. **** *p* < 0.005.

We utilized a combination of LASSO regression, SVM-RFE, and RF analysis to screen for synaptic plasticity-related hub genes in MDD from the 102 DEGs. Through LASSO regression analysis, seven key synaptic plasticity-related DEGs were identified (Figure 4A,B). Four key synaptic plasticity-related DEGs (Figure 4C) were identified using the SVM-RFE method. Using the RF algorithm, the top 30 genes were selected as key synaptic plasticity-related DEGs (Figure 4D,E) based on the mean decrease accuracy (MDA) and the mean decrease Gini (MDG). Finally, the synaptic plasticity-related DEGs detected with each method were intersected, resulting in the identification of two critical synaptic plasticity-related DEGs as hub genes (Figure 4F, Table S8). Then, the expression patterns of the hub genes between the MDD group and the control group were analyzed using box plots. The expression level of the hub gene CASKIN1 in the MDD group was significantly lower than that in the control group, while the expression level of the key gene CSTB in the MDD group was significantly higher than that in the healthy control group (Figure 4G).

### 2.7. Signaling Pathways Analysis

GSVA identified significant upregulation of 28 Hallmark signaling pathways in the MDD group, including HALLMARK_TNFA_SIGNALING_VIA_NFKB, HALLMARK_TGF_BETA_SIGNALING, and HALLMARK_REACTIVE_OXYGEN_SPECIES_PATHWAY (Figure S10, Table S9).

Correlation analysis between hub genes and Hallmark signaling pathways showed that CASKIN1 was negatively correlated with several pathways, including HALLMARK_TGF_BETA_SIGNALING, HALLMARK_PROTEIN_SECRETION, and HALLMARK_PI3K_AKT_MTOR_SIGNALING. CSTB was positively correlated with pathways such as HALLMARK_XENOBIOTIC_METABOLISM, HALLMARK_TNFA_SIGNALING_VIA_NFKB, and HALLMARK_REACTIVE_OXYGEN_SPECIES_PATHWAY (Figure S11).

### 2.8. GSEA of the DEGs

GSEA of DEGs between MDD and control samples in GSE38206 identified significant pathways (Table S10) based on the normalized enrichment score (NES), including LEISHMANIA INFECTION (NES = 2.7441), FC GAMMA R MEDIATED PHAGOCYTOSIS (NES = 2.4224), NOD LIKE RECEPTOR SIGNALING PATHWAY (NES = 2.3107), and CYTOKINE CYTOKINE RECEPTOR INTERACTION (NES = 1.3189), which were significantly enriched in the MDD group. STEROID HORMONE BIOSYNTHESIS (NES = −1.6192) and OLFACTORY TRANSDUCTION (NES = −1.8805) were significantly enriched in the healthy controls (Figure S12).

### 2.9. Interaction Network Analysis of the Hub Genes

**Figure 5:**
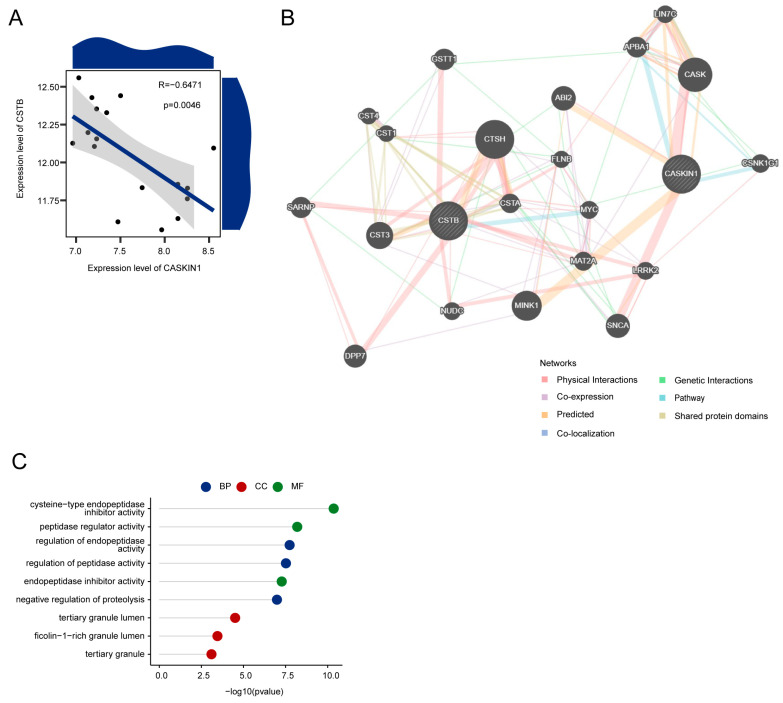
Interaction network analysis of hub genes. (**A**) Scatter plot showing the correlation between the expression levels of the two hub genes, illustrating the relationship between them in the dataset. (**B**) Gene co-expression network visualizing the interactions between the hub genes and their co-expressed genes, highlighting the network of connections that may contribute to synaptic plasticity in MDD. (**C**) GO enrichment analysis of co-expressed genes, displaying the biological processes, cellular components, and molecular functions that are significantly enriched among the genes in the co-expression network.

A significant negative correlation was observed between CASKIN1 and CSTB expression (Figure 5A). GeneMANIA database was utilized to construct an interaction network involving hub genes (Figure 5B). GO enrichment analysis in this network revealed significant enrichment in processes such as the regulation of endopeptidase activity, regulation of peptidase activity, and negative regulation of proteolysis; cellular components such as tertiary granule lumen, ficolin-1-rich granule lumen, and tertiary granule; and molecular functions including cysteine-type endopeptidase inhibitor activity. Additionally, molecular functions such as peptidase regulator activity and endopeptidase inhibitor activity were identified (Figure 5C, Table S11).

### 2.10. Construction of ceRNA, RBP, and TF Regulatory Networks

**Figure 6:**
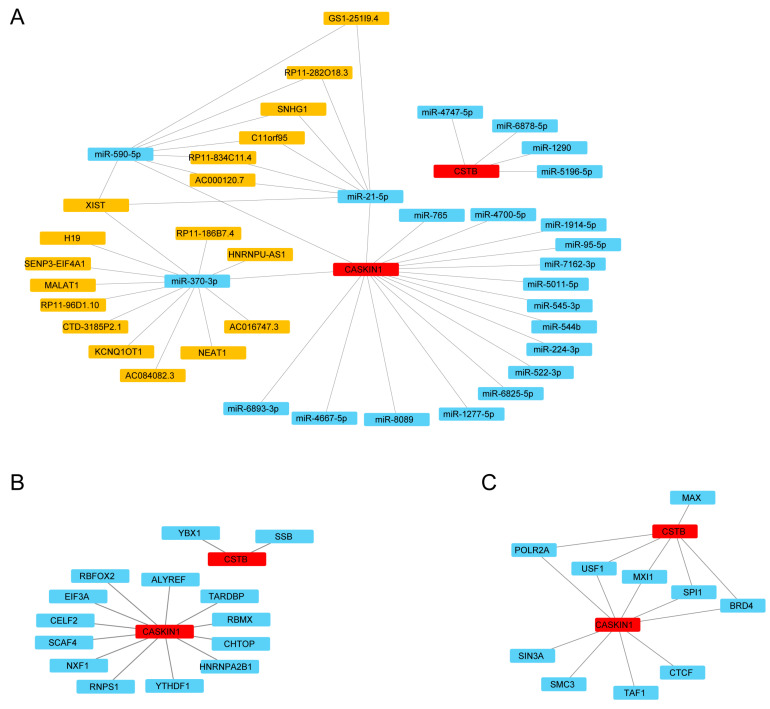
The regulatory network of ceRNAs, RBPs, and TFs. (**A**) The mRNA-miRNA-lncRNA network of hub genes, where red nodes represent hub genes, blue nodes represent microRNAs, and yellow nodes represent lncRNAs, depicting the interactions between these regulatory molecules. (**B**) The RBP-mRNA regulatory network of hub genes, with red nodes indicating hub genes and blue nodes representing RNA-binding proteins (RBPs), illustrating how RBPs interact with hub genes at the post-transcriptional level. (**C**) The mRNA-TF interaction network of hub genes, where red nodes signify hub genes and blue nodes denote transcription factors (TFs), showing the regulatory connections between hub genes and TFs that influence gene expression.

To elucidate the potential molecular mechanism of the hub genes in MDD, we constructed an interactive network of mRNA-miRNA-lncRNA. miR-21-5p was confirmed to target CASKIN1, with interacting lncRNAs including XIST, AC000120.7, RP11-834C11.4, C11orf95, SNHG1, RP11-282O18.3, and GS1-251I9.4 (Figure 6A).

Due to the interaction between RBPs and mRNA, we searched for and downloaded two mRNA/RBP pairs corresponding to hub mRNAs using the Starbase2.0 online database. Both hub genes had corresponding pairing information. Based on the relationship between target genes provided by online datasets, an RBP-mRNA network was constructed, consisting of 16 nodes (14 RBPs and 2 mRNAs) as depicted in (Figure 6B). Details of nodes and interactions are listed in Table S12.

We searched for TFs binding to the hub genes through the hTFtarget database and obtained interaction data for 2 hub genes and 10 TFs, which were visualized using Cytoscape v 3.10.0 (National Human Genome Research Institute, Bethesda, MD, USA) (https://cytoscape.org/ (accessed on 3 June 2024)) (Figure 6C). The mRNA-TF interaction network reveals numerous interactions involving the hub gene CASKIN1 and TFs. A total of nine pairs of mRNA-TF interactions were identified, as detailed in Table S13.

## 3. Discussion

This study focuses on the differential gene expression and underlying mechanisms of MDD by comparing MDD patients to controls. By integrating single-nucleus sequencing data and whole-genome expression profiles, this study aimed to uncover the molecular mechanisms associated with MDD. The comprehensive analysis highlights the potential to identify key genes and regulatory networks, offering new insights for future diagnostic and therapeutic approaches.

Using snRNA-seq data and bioinformatics analysis methods, we investigated cell type-specific gene expression patterns in MDD, focusing on synaptic plasticity. Our findings indicate significant changes in Excitatory.neurons_1 cells related to synaptic plasticity and MDD. We identified CASKIN1 and CSTB as hub genes closely associated with the mechanisms underlying synaptic plasticity in MDD.

We analyzed 78,886 cells from single-nucleus transcriptomes, identifying 9 cell types and 21 clusters through clustering and biomarker annotation. Compared to Nagy’s study [24], our analysis identified a previously unrecognized cortical cell cluster and fewer cell clusters (21 vs. 26), which may be attributed to differences in clustering and annotation methodologies, as well as the use of updated gene expression profiles for cell-type identification. Notably, oligodendrocyte precursor cells (OPCs) and excitatory neurons showed the most significant changes in proportion between the control and MDD groups. In previous studies of the same dataset, it was found that one cluster, composed of immature OPCs (OPC2), and another, made up of deep-layer excitatory neurons (Ex7), accounted for nearly half (47%) of the dysregulated genes, highlighting the critical role of OPCs and excitatory neurons in MDD [24,25]. Further studies have also confirmed that the proliferation capacity of OPCs decreases in the MDD group, which may explain the reduced proportion of these cells in MDD patients [26].

We identified 18,055 cells with high synaptic plasticity-related gene activity. According to the UMAP diagram, most cell subsets of Excitatory.neurons_1 exhibited high synaptic plasticity activity. Based on the synaptic plasticity scores, Excitatory.neurons_1 cells showed a higher score and a larger difference between the control and MDD groups, so we focused more on Excitatory.neurons_1 cells. Research has shown that genes related to excitatory synaptic function are significantly associated with depression phenotypes [27]. Previous research has also indicated that excitatory neurons play a crucial role in synaptic plasticity, particularly in long-term potentiation (LTP) and long-term depression (LTD) [28]. In our study, the proportion of Excitatory.neurons_1 cells increased in the MDD group, with reduced synaptic plasticity activity, suggesting potential alterations in synaptic plasticity that may contribute to impaired learning and memory functions in MDD patients [29].

Pseudotime trajectory analysis of cells with high synaptic plasticity activity revealed three distinct branches with unique differentiation patterns. Among these, the Cell fate 1 state showed functional enrichment in axon and neuron guidance, while the Cell fate 2 state showed enrichment in synaptic regulation. The increased proportion of MDD cells and Excitatory.neurons_1 cells in Cell fate 2 underscores their association with synaptic plasticity and MDD.

The communication analysis revealed a reduction in both the number and intensity of interactions in the MDD group compared to the control group, indicating decreased cellular activity in the MDD group. Through ligand-receptor pairs analysis involving Excitatory.neurons_1 cells and other cell clusters, we identified the significant roles of NRG and CNTN signaling pathways in the interactions between Excitatory.neurons_1 cells and OPCs_1, Macrophage.microglia cells, and Inhibitory.neurons_3 cells. Both gene families, NRG and CNTN, are known to play crucial roles in processes like synaptic formation and neuronal migration [30,31], suggesting potential influencing factors of Excitatory.neurons_1 cells in synaptic plasticity alterations.

Using a combination of snRNA-seq and bulk RNA-seq data, we identified 102 DEGs related to synaptic plasticity in the Excitatory.neurons_1 cells of MDD. GO term enrichment analysis for these DEGs revealed significant involvement in processes such as axon extension and neuron projection extension, indicating disruptions in neural connectivity in the MDD group. KEGG pathway analysis highlighted the involvement of cardiomyopathy-related pathways, suggesting potential systemic effects of MDD on cardiovascular health.

Using multiple machine-learning approaches, we identified CASKIN1 and CSTB as hub genes of synaptic plasticity in MDD. CASKIN1, a scaffold protein crucial for synaptic signaling, forms part of the fibrous protein network that structures the active zones of neural synapse [32]. According to the Human Protein Atlas (https://www.proteinatlas.org/ENSG00000167971-CASKIN1/brain (accessed on 20 March 2025)), CASKIN1 exhibits tissue-enriched expression in the brain. Research using CASKIN1 knockout mice revealed its widespread distribution in both the brain and spinal cord, predominantly at synapses [33], suggesting its critical role in synaptic function. In our analyzed datasets, the expression of CASKIN1 was found to be downregulated in the MDD group. Studies have shown that CASKIN1 and CASKIN2 knockout mice exhibited reduced synaptic profiles and dendritic spines in hippocampal CA1 pyramidal neurons, specifically altered AMPA receptor phosphorylation, and impaired LTP induction in hippocampal slices. This indicates that the loss of Caskins leads to severe deficits in novelty recognition and spatial memory [34]. Thus, the decrease in CASKIN1 may lead to a loss of synaptic integrity, potentially contributing to the cognitive decline observed in MDD by affecting the regulation of LTP in Excitatory.neurons_1 cells. Conversely, CSTB, a cysteine protease inhibitor [35], was upregulated in MDD in our analyzed datasets. In situ hybridization and immunocytochemistry studies on adult rat brains detected CSTB in virtually all forebrain neurons but not in glial cells. And it was discovered that epileptic seizures rapidly increase CSTB synthesis in forebrain neurons [36], suggesting a link between CSTB and neural activity. Moreover, research has indicated that CSTB exists in neuronal synaptic regions [37] and is secreted by synaptosomes, with membrane depolarization enhancing this secretion. This finding suggests that the secretion of CSTB at synapses is regulated by synaptic plasticity activity, implying that the increased levels of CSTB in the MDD group may reflect a compensatory response to neuroinflammation and cellular stress.

GSEA and GSVA further elucidated dysregulated molecular pathways in MDD, highlighting the roles of inflammation and oxidative stress. Pathways such as TNF-α signaling via NF-κB, TGF-β signaling, and reactive oxygen species pathways were significantly upregulated, reinforcing their importance in MDD pathophysiology [38,39].

The interaction network encompassing CASKIN1, CSTB, and their associated genes revealed a complex interplay involving 22 genes. GO enrichment analysis underscored the multifaceted roles of these genes in cellular regulatory mechanisms implicated in MDD. Regulatory network analyses provide a comprehensive framework for understanding the molecular underpinnings of MDD, identifying miR-21-5p as a critical regulator targeting CASKIN1. Dong et al. [40] conducted a dual-luciferase reporter assay to confirm the direct binding of miR-21-5p to the 3′UTR of CASKIN1, as evidenced by reduced luciferase activity in the wild-type 3′UTR but not in a mutant version. Additionally, correlation analysis in low-grade glioma (LGG) patients revealed a negative relationship between miR-21-5p and CASKIN1 expression, while Western blot results showed that miR-21-5p overexpression decreased CASKIN1 protein levels, and its inhibition increased CASKIN1 expression. Similarly, Lv’s study [41] validated the regulation of CASKIN1 by miR-21a-5p through dual-luciferase assays, qRT-PCR, and Western blot analysis. Based on this evidence, we hypothesize that the downregulation of CASKIN1 in MDD may be attributed to elevated miR-21-5p levels.

Based on the above findings, we propose that elevated miR-21-5p levels in MDD lead to the downregulation of CASKIN1 in Excitatory.neurons_1 cells, resulting in decreased neural connectivity and altered synaptic plasticity. This impairs LTP and weakens the long-term enhancement of synaptic transmission, contributing to cognitive decline. Conversely, in response to changes in synaptic plasticity, CSTB expression increases, suggesting its role in modulating these effects. However, these hypotheses require further experimental validation.

The snRNA-seq dataset GSE144136 analyzed in our study was originally generated by Nagy et al. [24]. Their study focused on cell-type-specific transcriptomic alterations in MDD and identified hub genes through WGCNA. In contrast, our study specifically investigated the role of synaptic plasticity in MDD, aiming to identify key cell clusters, hub genes, and their regulatory mechanisms. To achieve this, we integrated snRNA-seq and bulk RNA-seq data, employing multiple machine-learning approaches for hub gene identification. Machine learning was chosen due to its superior feature selection capabilities, allowing for more precise identification of disease-related genes in complex datasets. While WGCNA is effective at detecting co-expression patterns and identifying highly connected genes in network modules, machine learning focuses on genes with the highest predictive power for distinguishing MDD from healthy controls, with the added benefit of cross-validation to enhance the robustness and reliability of predictions. Nagy et al. identified gene expression alterations in oligodendrocyte precursor cells and deep-layer excitatory neurons [24]. Using WGCNA, they detected 285 hub genes, and the top 50 included 10 MDD-related DEGs involved in neurotransmitter secretion and synaptic plasticity. In comparison, our study identified Excitatory.neurons_1 as the most relevant cell cluster associated with synaptic plasticity in MDD. Additionally, through machine learning, we identified CASKIN1 and CSTB as novel hub genes linked to synaptic plasticity in MDD genes that were not among the 10 MDD-related hub genes selected by Nagy et al. This comparison demonstrates that machine-learning methods can uncover novel insights by analyzing gene expression from a different perspective, providing complementary insights into the molecular mechanisms of MDD.

Several limitations should be acknowledged. First, although we identified hub genes by intersecting DEGs from GSE144136 and GSE38206 datasets and used multiple machine learning methods with rigorous cross-validation, our results lack external validation, which is essential to confirm the biological relevance of these hub genes and to determine whether they are dataset-specific or potential artifacts. In the future, we plan to incorporate independent datasets for further validation. Second, our study relied solely on computational methods without validation through wet-lab experiments. In particular, the experimental validation of hub gene expression in MDD brain tissues is essential, and our future research will include wet-lab experiments to provide more evidence and strengthen the reliability of our findings. Third, the sample size was relatively small, potentially limiting the generalizability of the results. Additionally, the use of multiple datasets introduces the possibility of batch effects, despite efforts to correct for these.

While prior studies of MDD at the single-nucleus level have predominantly focused on oligodendrocytes [26,42,43], with limited attention on excitatory neurons, our study identified Excitatory.neurons_1 as a potentially significant cell cluster associated with synaptic plasticity in MDD. However, it is important to note that the snRNA-seq dataset used in our study only included male samples. Previous studies have demonstrated that deep-layer excitatory neurons, astrocytes, and oligodendrocyte precursors are the main contributors to DEGs in males, whereas microglia and intermediate oligodendrocytes are the major contributors in females in MDD [25]. This suggests that MDD may exhibit gender-specific expression differences in certain cell types and genes, indicating that depression in males and females may have distinct molecular mechanisms. Therefore, our findings may be more relevant to males, and further research including female samples will be needed to explore the impact of sex differences on the molecular mechanisms underlying synaptic plasticity in MDD.

## 4. Materials and Methods

### 4.1. Single-Nucleus RNA-Seq Data Processing

The single-nucleus RNA-seq dataset GSE144136 was obtained from the Gene Expression Omnibus (GEO) database using the “GEOquery” R package version 4.1.2 (https://www.r-project.org/ (accessed on 10 May 2024)) and processed with the “Seurat” R package. This dataset contains 34 postmortem dorsolateral prefrontal cortex samples from 17 individuals with MDD and 17 healthy controls, all of whom were male. After normalization, highly variable genes were identified by balancing the average expression and dispersion. Principal component analysis (PCA) was then performed, and significant principal components (PCs) were used for graph-based clustering. Batch effects were removed using “RunHarmony”, with the batch group set to each sample. Cell clustering was executed with the “FindClusters” function, followed by the “RunUMAP” function for uniform manifold approximation and projection (UMAP) to visualize cell clustering. Differentially expressed genes (DEGs) in each cell cluster were identified using the “FindAllMarkers” function. Subsequently, cell clusters were annotated using cell type-specific biomarkers, referred to as the cell marker information provided in the previous research [24], and the proportion of each cell cluster was evaluated.

### 4.2. Identification of Synaptic Plasticity Activity in Different Cell Subsets

Synaptic plasticity-related genes used for analysis came from the MSIGDB database (https://www.gseamsigdb.org/gsea/msigdb/human/geneset/GOBP_LONG_TERM_SYNAPTIC_DEPRESSION (accessed on 22 May 2024)). Synaptic plasticity-related genes in each nucleus were analyzed using the “AUCell” R package based on GSEA [44]. The area under the curve (AUC) values for 182 synaptic plasticity-related genes (Table S1) were used to estimate the proportion of highly expressed gene sets. And the “AUCell_exploreThresholds” function determined the optimal threshold for identifying activated cell subsets. The “ggplot2” R package was then employed to map the AUC scores onto a UMAP embedding, visualizing activated cell subsets.

### 4.3. Pseudotime Trajectory Analysis

Pseudotime trajectories were constructed using genes with high dispersion and expression (discrete estimated value ≥ 1 and average expression ≥ 0.1) with the “Monocle2” R package. Branch expression analysis modeling (BEAM) in Monocle2 identified genes with significant branch-dependent expression [45], which were then visualized using heat maps.

### 4.4. Cell–CellCommunication and Ligand-Receptor Interaction Analysis

A CellChat object was generated using the “CellChat” R package, utilizing the unique molecular identifiers (UMIs) count matrices from the MDD and the control groups. The intercellular communication and ligand–receptor interaction analysis were analyzed using the “CellChatDB.human” database. CellChat objects from both groups were integrated using the “mergeCellChat” function to compare interaction numbers and intensities. The “netVisual_diffInteraction” function visualized differences in interaction numbers or intensities across cell clusters, while “netVisual_bubble” and “netVisual_aggregate” functions illustrated the signal gene expression distribution between groups.

### 4.5. Bulk RNA-Seq Data Processing

Bulk RNA-seq dataset GSE38206 was obtained from the GEO database, comprising blood samples from 9 MDD patients and 9 healthy controls. The batch effect was corrected using the “ComBat method” (the “sva” R package), and PCA assessed the correction’s effectiveness.

### 4.6. Differential Expression Analysis

DEGs between Excitatory.neurons_1 and other cell clusters were identified using the “Seurat” R package with criteria |log2FoldChange| > 0.25 and *p* < 0.05. DEGs between the MDD and control groups in GSE38206 were identified using the “limma” R package with criteria |log2FoldChange| > 0.5 and adjusted *p* < 0.05. Synaptic plasticity in Excitatory.neurons_1 cells from MDD were identified by intersecting these DEG sets.

### 4.7. Machine Learning Analysis

Least absolute shrinkage and selection operator (LASSO) regression (the “glmnet” R package), random forest (RF) analysis (the “RandomForest” R package), and the support vector machine-recursive feature elimination (SVM-RFE) method (the “e1071” R package) were combined to identify the hub genes. For the SVM model, 10-fold cross-validation (nfold = 10) was employed to assess model performance. In the LASSO regression model, 100 different λ values were used along the regularization path (nlambda = 100), with L1 regularization (i.e., Elastic Net L1 regularization, alpha = 1). Additionally, 10-fold cross-validation (nfolds = 10) was applied for model selection and evaluation. For the random forest model, the randomForest function was used to build the model, specifying the generation of 1000 trees (ntree = 1000) to enhance model stability and predictive power.

### 4.8. Functional Enrichment Analysis

GSEA analyzed DEGs between the MDD and control groups in GSE38206 using the “clusterProfiler” R package with 1000 gene set permutations per analysis. c2.cp.kegg.v7.5.1.symbols from the MSigDB was the reference gene set [46], with gene sets considered significantly enriched if *p* < 0.05. Gene set variation analysis (GSVA) analyzed differences in 50 Hallmark signaling pathways between MDD and controls, correlating hub genes with these pathways using the “GSVA” R package. GO and KEGG enrichment analyses were conducted using the “clusterProfiler” R package.

### 4.9. Co-Expressed Genes Interaction Network Construction

The interaction network of hub genes and the co-expressed genes was constructed using GeneMANIA (http://genemania.org (accessed on 3 June 2024)). GO enrichment analysis explored the functions of hub genes and their interacting genes.

### 4.10. CeRNA Network Construction

Starbase2.0 (https://starbase.sysu.edu.cn/tutorialAPI.php#RBPTarget (accessed on 23 May 2024)) and miRDB (https://mirdb.org/index.html (accessed on 23 May 2024)) databases were used to predict potential microRNAs targeting hub genes. LncRNAs interacting with these microRNAs were identified to construct the competitive endogenous RNA (ceRNA) network.

### 4.11. RBP-mRNA Network Construction

Starbase2.0 analyzed ncRNA interactions. The relationship between mRNA and RNA binding protein (RBP) expression was investigated using CLIP-seq, degradome-seq, and RNA-RNA interaction data. Criteria for identifying key mRNA-RBP pairs in disease contexts were *p* < 0.05, clusterNum ≥ 5, and clipExpNum ≥ 5. The RBP-mRNA network was constructed using Cytoscape v 3.10.0 (https://cytoscape.org/ (accessed on 3 June 2024)).

### 4.12. mRNA-TF Network Construction

Transcription factors (TFs) and their target genes in brain tissues were obtained from the hTFtarget database (http://bioinfo.life.hust.edu.cn/hTFtarget#! (accessed on 3 June 2024)). Target genes were screened using hub genes in this study, and the mRNA-TF network was constructed using Cytoscape.

### 4.13. Statistical Analysis

Statistical analysis was performed using R software (version 4.1.2). Spearman’s correlation assessed the correlation between parameters, and the Wilcoxon test compared differences between groups. A significance level of *p* < 0.05 was considered statistically significant.

## 5. Conclusions

In conclusion, this study provides valuable insights into the mechanisms of MDD from the perspective of synaptic plasticity at the single-cell level. By identifying key molecules such as miR-21-5p, CASKIN1, and CSTB, our findings offer new biomarkers for the molecular mechanism, diagnosis, and treatment of MDD. Future studies should aim to validate these findings through experimental approaches and explore gender-specific differences to enhance our understanding of MDD.

## Data Availability

The datasets analyzed during the current study (GSE144136 and GSE38206) are available in the GEO database (https://www.ncbi.nlm.nih.gov/geo/ (accessed on 22 May 2024)). The data for bioinformatic analysis were previously generated and can be found in MSIGDB (https://www.gsea-msigdb.org/gsea/msigdb/human/geneset/GOBP_LONG_TERM_SYNAPTIC_DEPRESSION (accessed on 22 May 2024)), GeneMANIA (http://genemania.org (accessed on 3 June 2024)), Starbase2.0 (https://starbase.sysu.edu.cn/tutorialAPI.php#RBPTarget (accessed on 23 May 2024)), miRDB (https://mirdb.org/index.html (accessed on 23 May 2024)), Cytoscape v 3.10.0 (https://cytoscape.org/ (accessed on 3 June 2024)), and the hTFtarget database (http://bioinfo.life.hust.edu.cn/hTFtarget# (accessed on 3 June 2024)).

## Supplementary Materials

The following supporting information can be downloaded at: https://www.mdpi.com/article/10.3390/ijms26073135/s1.

## Abbreviations

The following abbreviations are used in this manuscript:

MDD	Major depressive disorder
HPA	Hpothalamic–pituitary–adrenal
BDNF	Bain-derived neurotrophic factor
mTOR	Mammalian target of rapamycin
SSRIs	Selective serotonin-reuptake inhibitors
TCAs	Tricyclic antidepressants
scRNA-seq	Single-cell RNA sequencing
snRNA-seq	Single-nucleus RNA sequencing
GO	Gene ontology
KEGG	Kyoto encyclopedia of genes and genomes
GSEA	Gene set enrichment analysis
GEO	Gene set enrichment analysis
PCA	Principal component analysis
PCs	Principal components
UMAP	Uniform manifold approximation and projection
DEGs	Differentially expressed genes
AUC	Area under the curve
BEAM	Branch expression analysis modeling
UMIs	Unique molecular identifiers
LASSO	Least absolute shrinkage and selection operator
RF	Random forest
SVM-RFE	Support vector machine–recursive feature elimination
GSVA	Gene set variation analysis
RBP	RNA binding protein
TFs	Transcription factors
MDG	Mean decrease Gini
MDA	Mean decrease accuracy
NES	Normalized enrichment score
LTP	Long-term potentiation
LTD	Long-term potentiation
