# Supplementary material for: Exploring Male-Specific Synaptic Plasticity in Major Depressive Disorder: A Single-Nucleus Transcriptomic Analysis Using Bioinformatics Methods
Source: Int J Mol Sci. 2025 Mar 28;26(7):3135. doi: 10.3390/ijms26073135 (PMC11989135; doi:10.3390/ijms26073135)
Supplement: Supplementary file 1 [file ijms-26-03135-s001.zip › Supplementary Figures.pdf]

Supplementary Figures

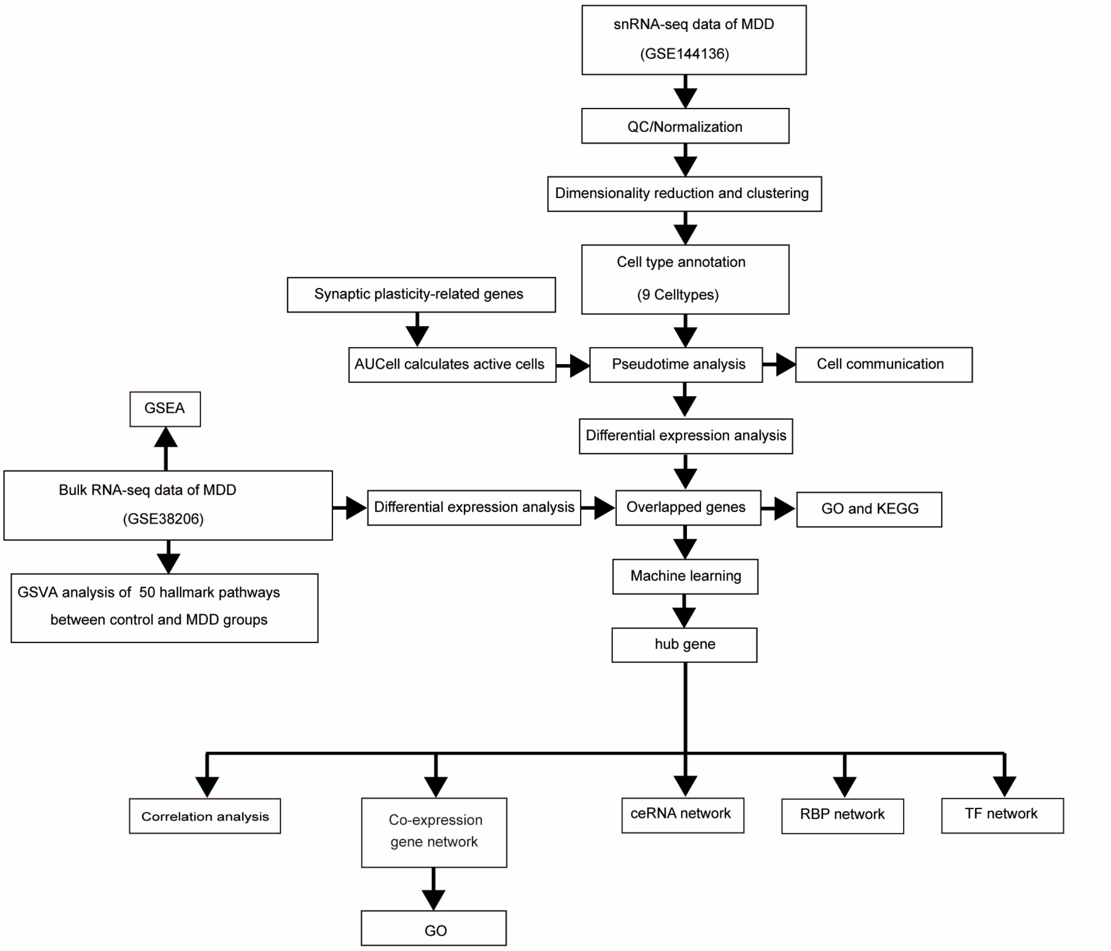

Figure S1. Workflow of this study.

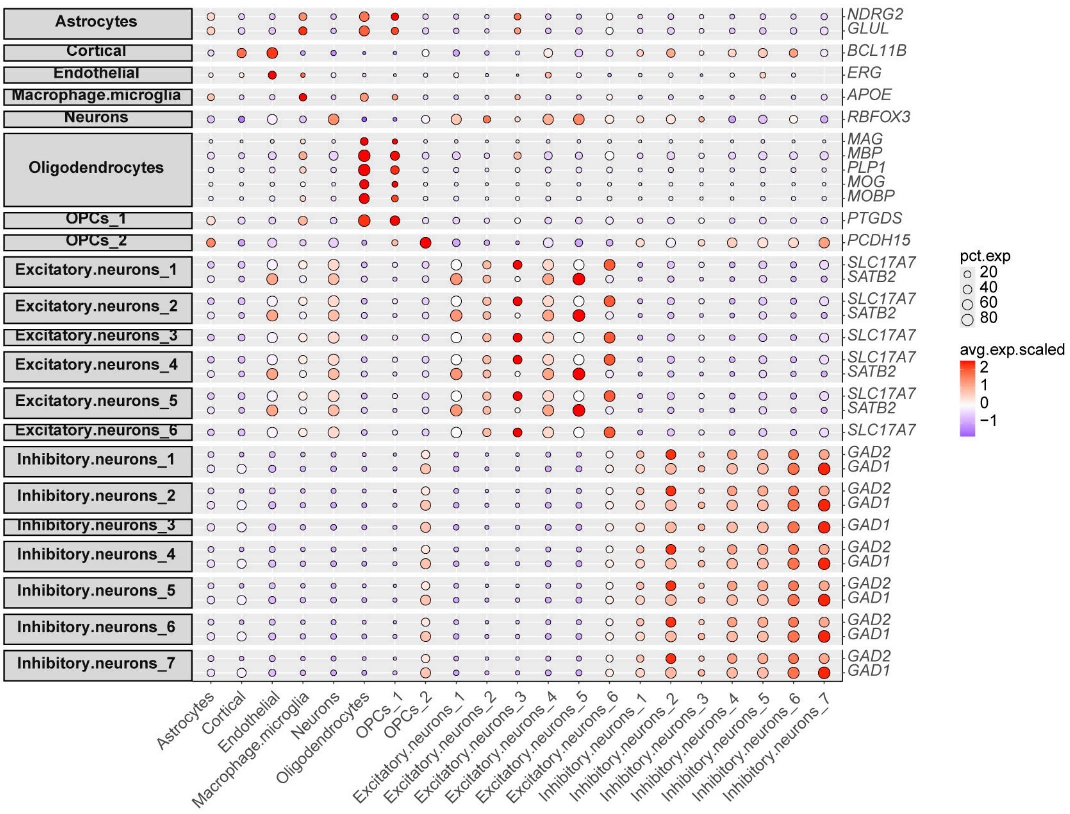

Figure S2. The expression of marker genes in each cell cluster.

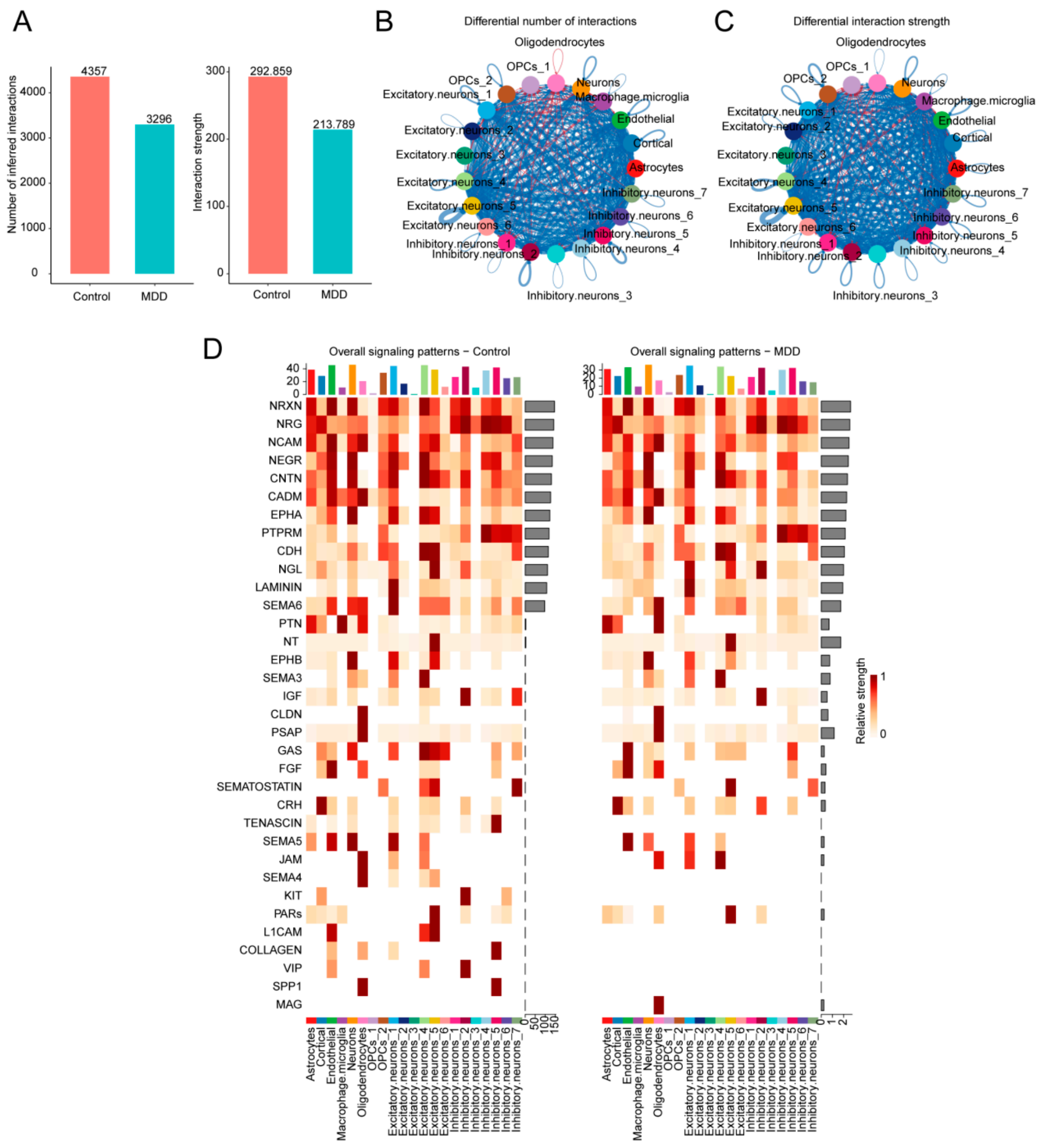

Figure S3. The cell-cell communication analysis.

(A) The number and intensity of cell-cell interactions between the control and the MDD groups.

(B) The network diagram of the number of interactions between cells.

(C) The network diagram of the intensity of interactions between cells.

(D) The heat map of the overall signaling patterns in the control and the MDD groups.

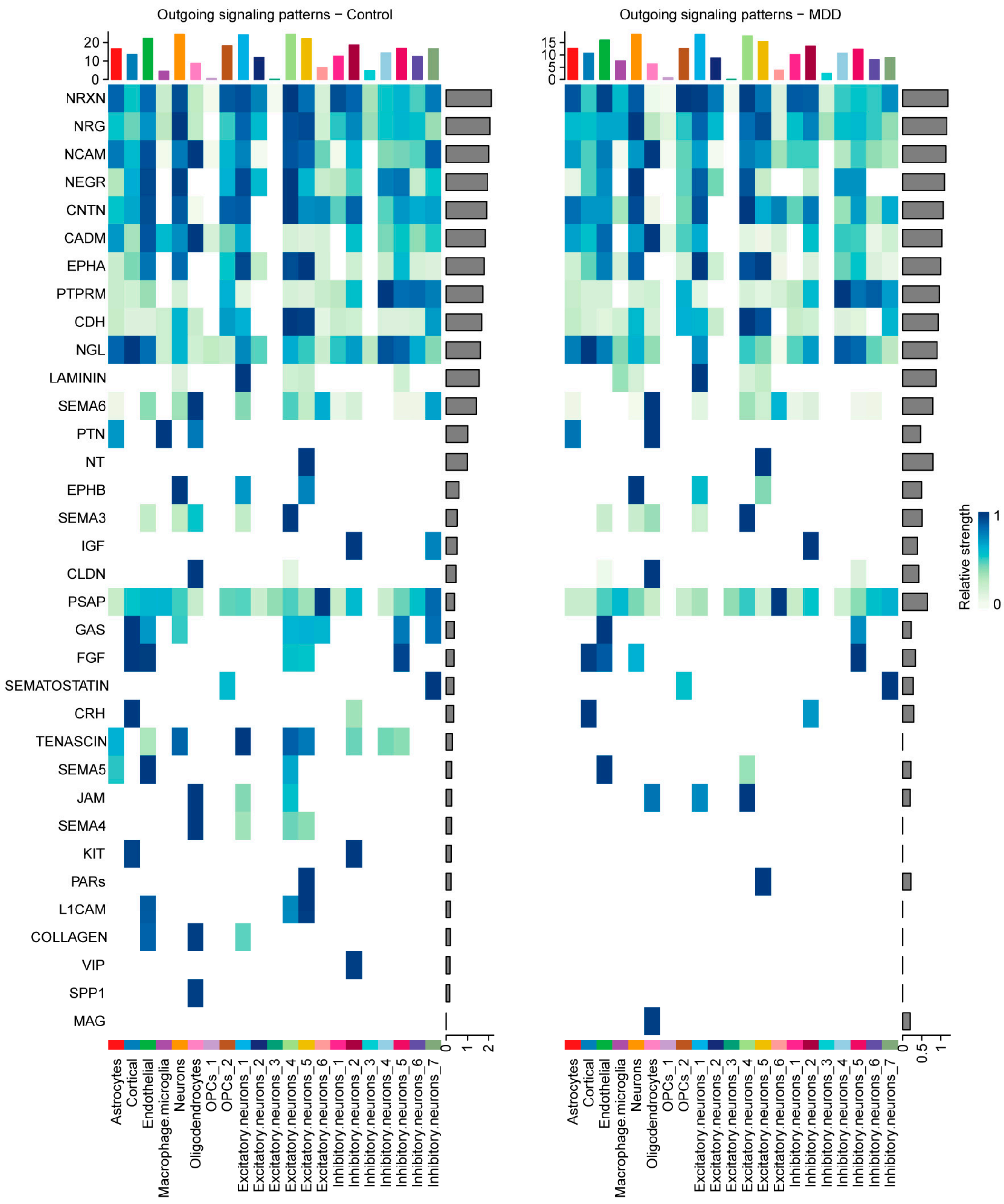

Figure S4. The heat map of the outgoing signaling patterns in the control and the MDD groups.

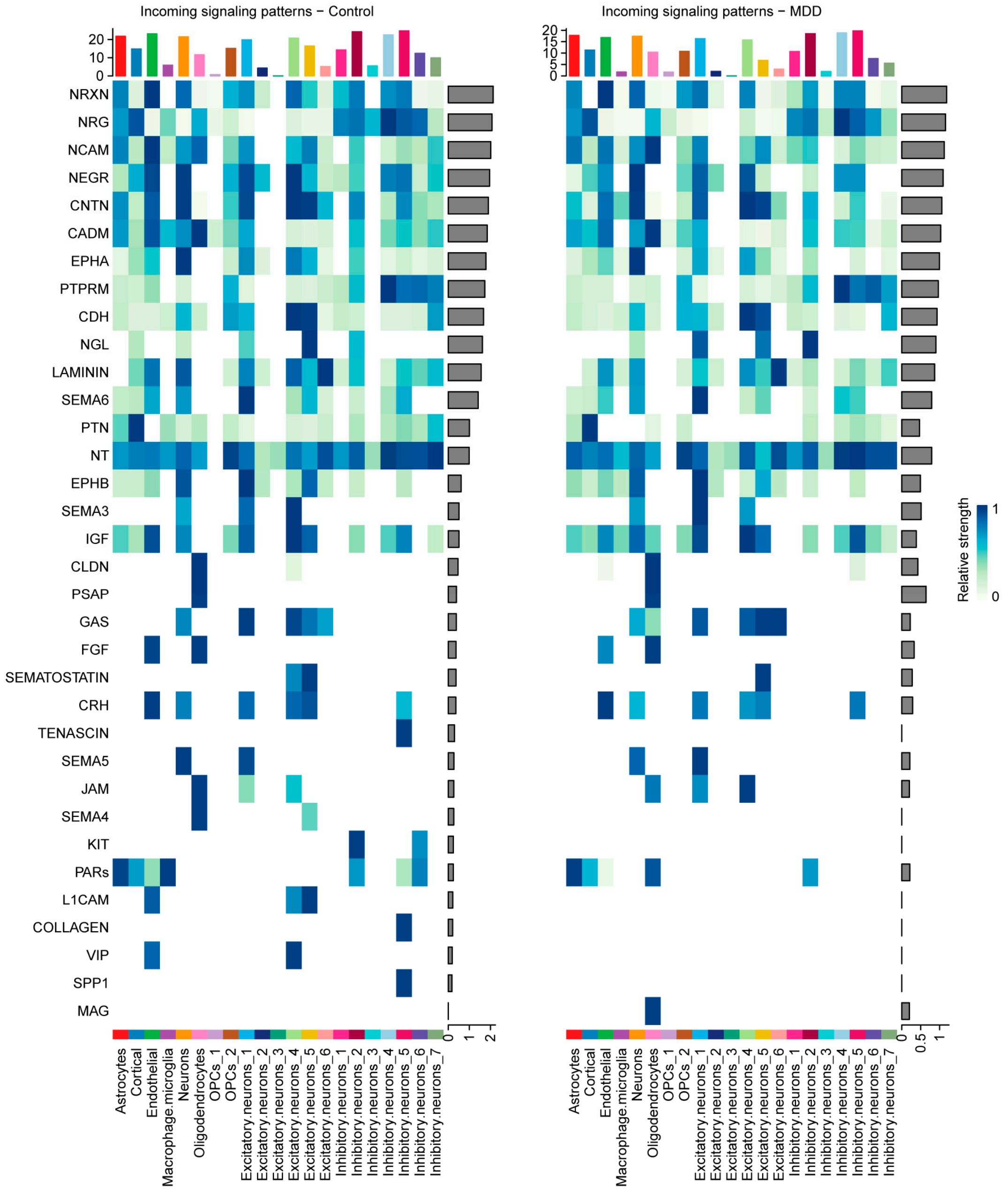

Figure S5. The heat map of the incoming signaling patterns in the control and the MDD groups.

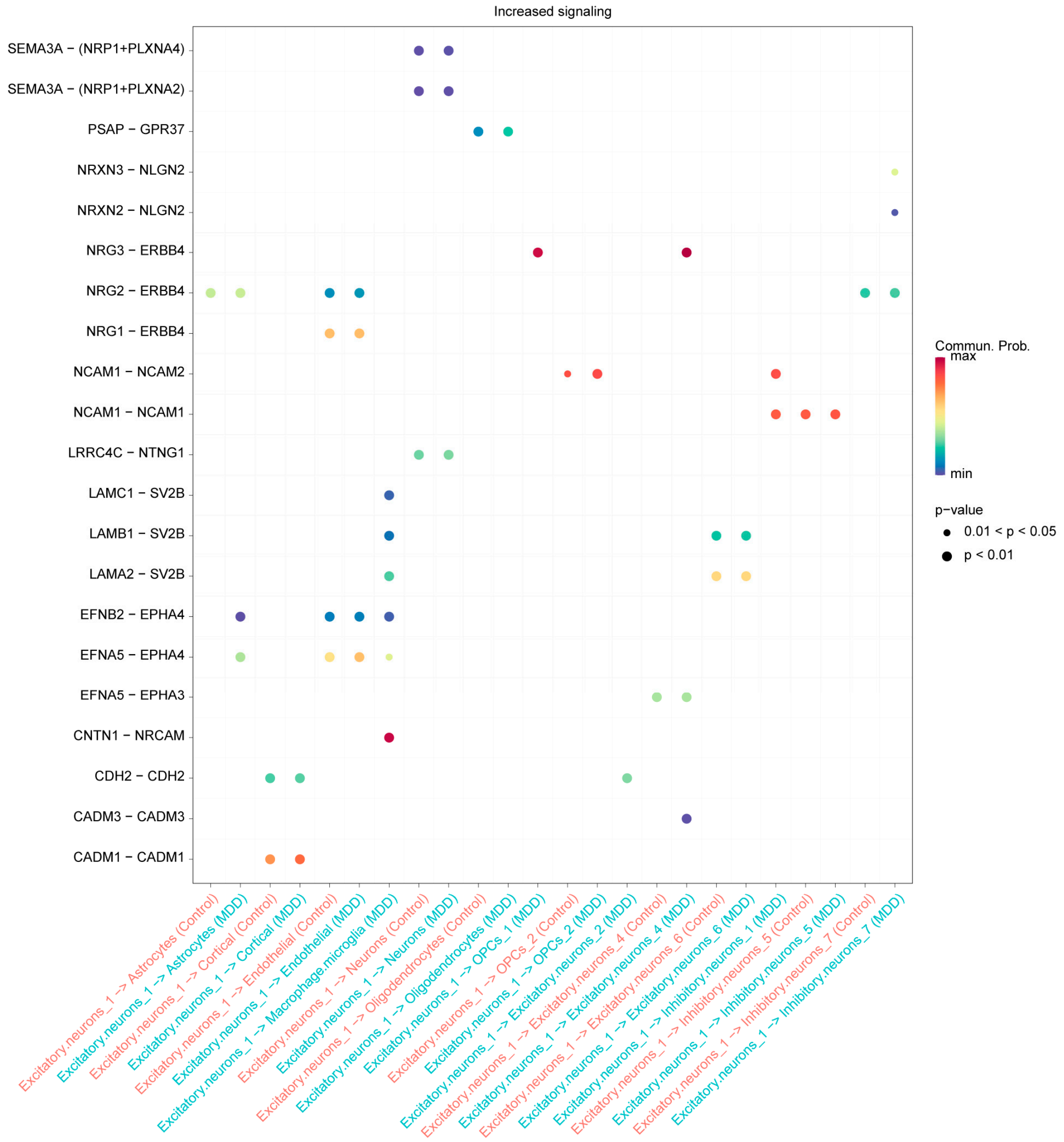

Figure S6. Ligand–receptor pairs with increased interaction intensity in the MDD group between Excitatory.neurons_1 cells and other cell clusters.

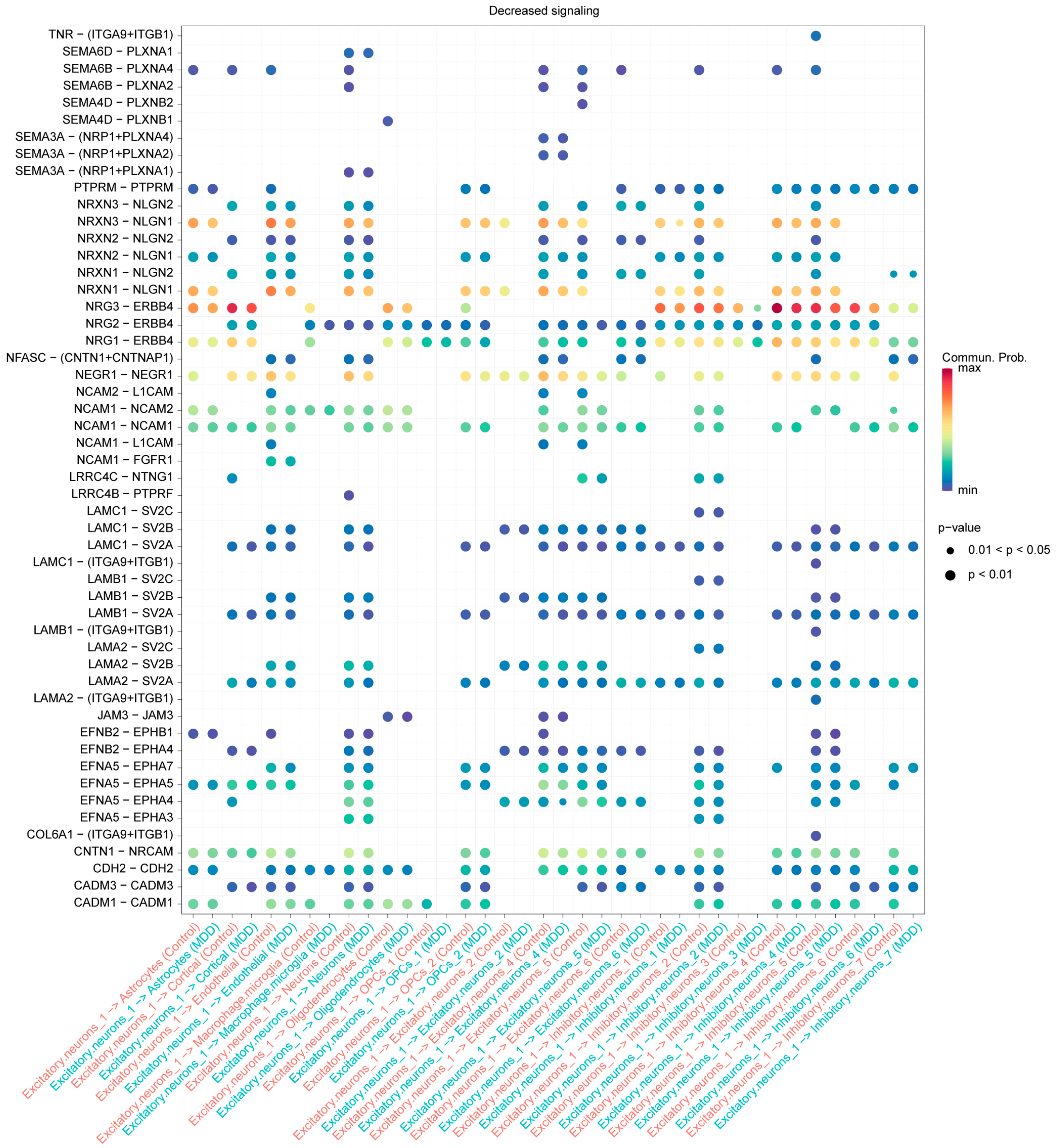

Figure S7. Ligand–receptor pairs with reduced interaction intensity in the MDD group between Excitatory.neurons_1 cells and other cell clusters.

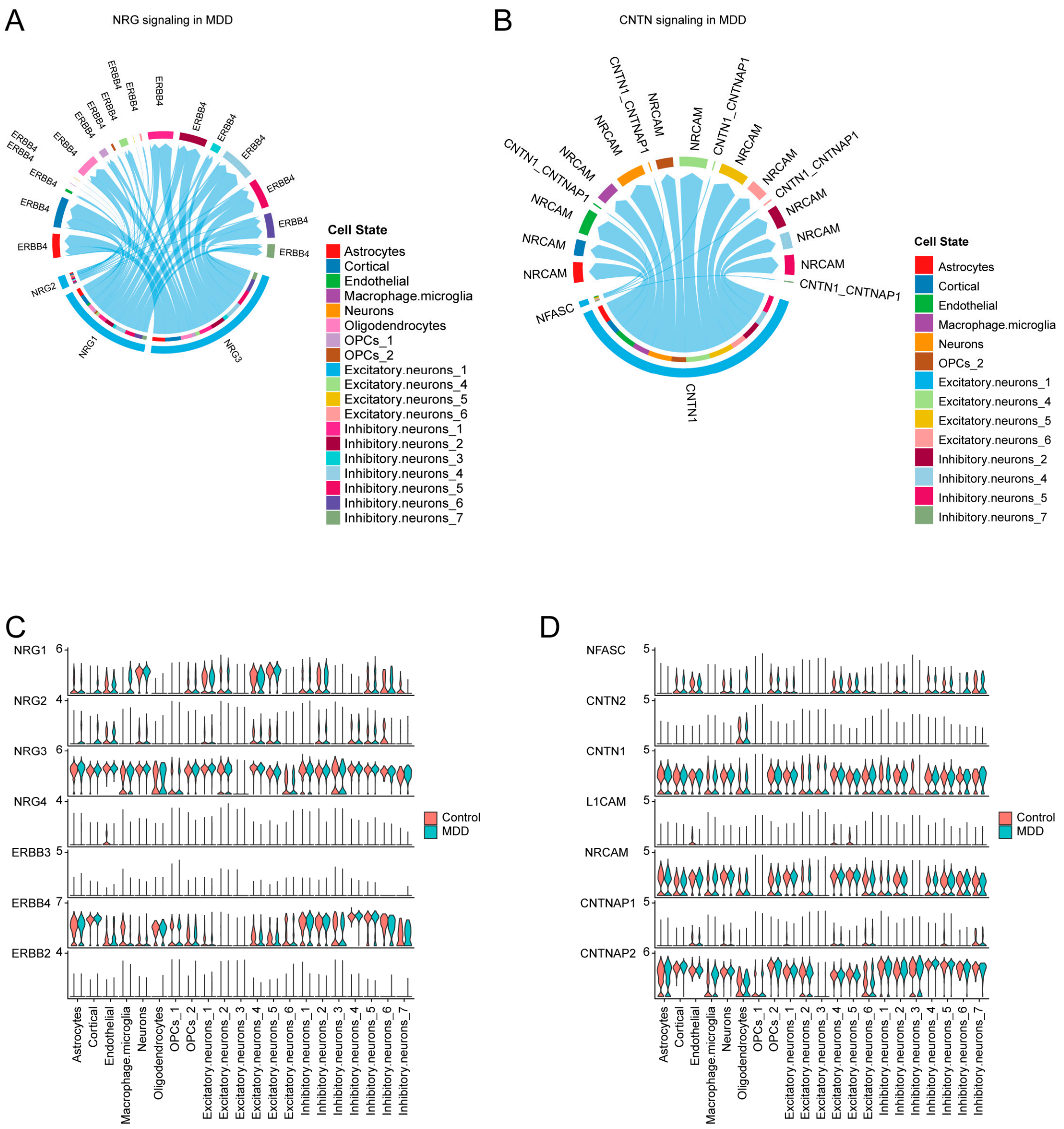

Figure S8. Ligand-receptor pairs in key signaling pathways.

(A) The ligand-receptor pairs that may regulate the communication between Excitatory.neurons_1 cells and other cells in NRG signaling pathway in the MDD group.

(B) The ligand-receptor pairs that may regulate the communication between Excitatory.neurons_1 cells and other cells in CNTN signaling pathway in the MDD group.

(C) The expression of NRG signaling ligand-receptor pairs in the control and

the MDD groups.

(D) The expression of CNTN ligand-receptor pairs in the control and the MDD groups.

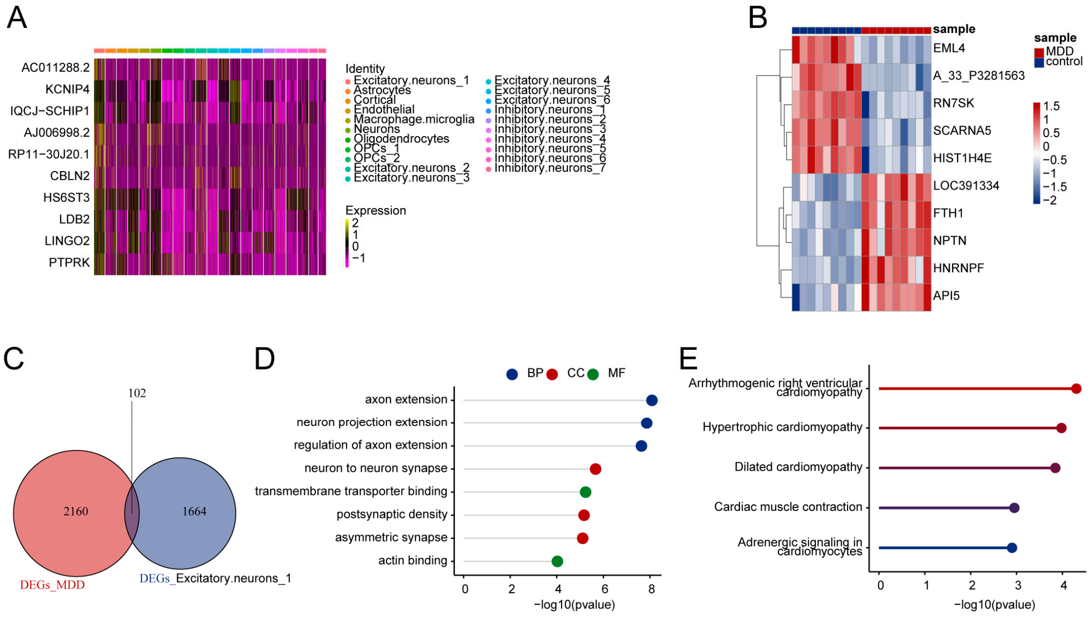

Figure S9. Analysis of synaptic plasticity-related DEGs in MDD.

(A) The heat map of 10 genes significantly up-regulated in Excitatory.neurons_1 cells in MDD.

(B) The heat map of 10 genes that are significantly differentially expressed in MDD. (C) The Venn diagram of the synaptic plasticity-related DEGs in MDD.

(C) GO enrichment analysis of the overlapping DEGs.

(E) KEGG enrichment analysis of the overlapping DEGs.

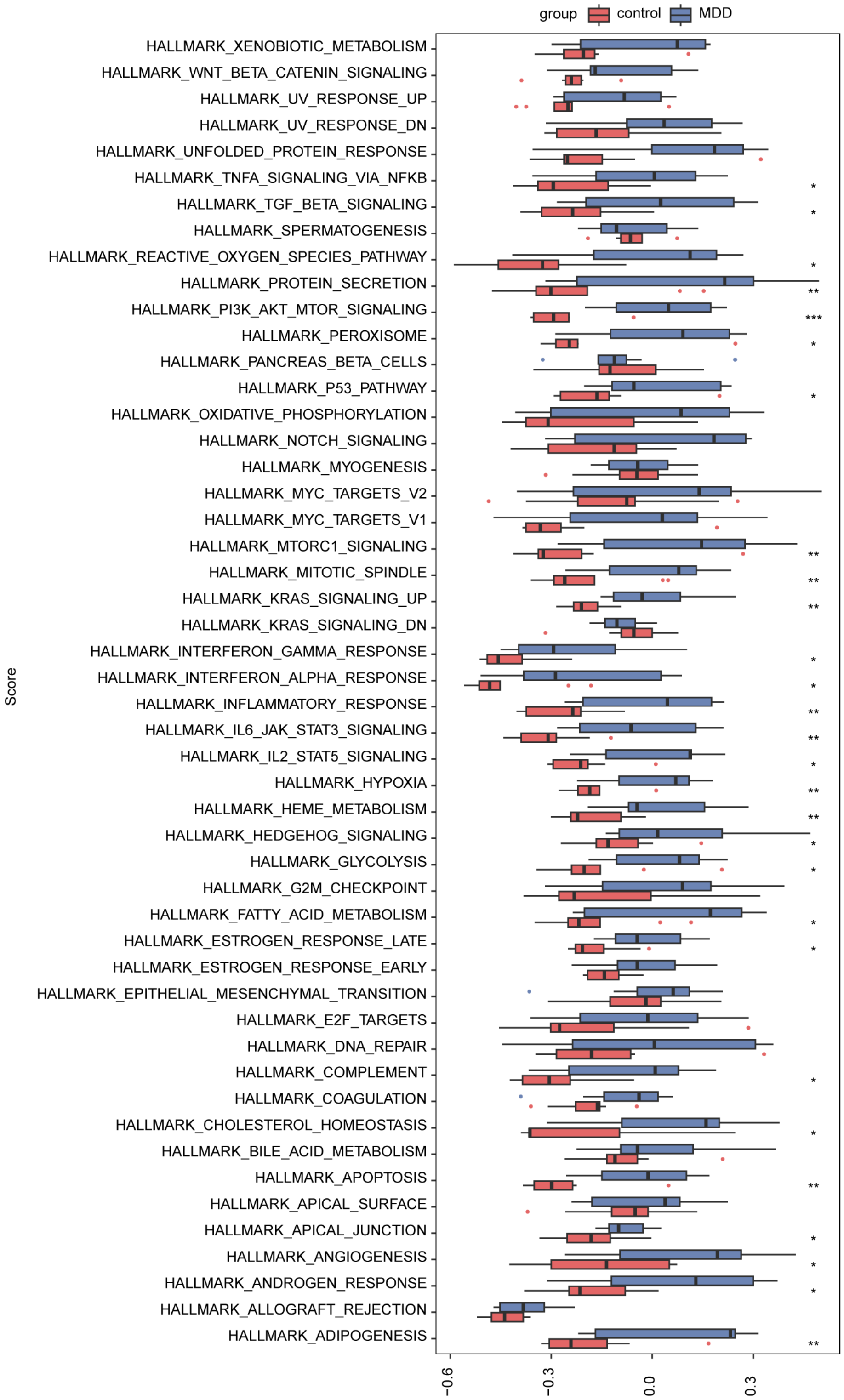

Figure S10. Comparison of 50 HALLMARK signaling pathways between the MDD group and the control group. ****$P < 0.0001$, ***$P < 0.001$, **$P < 0.01$, *$P < 0.05$.

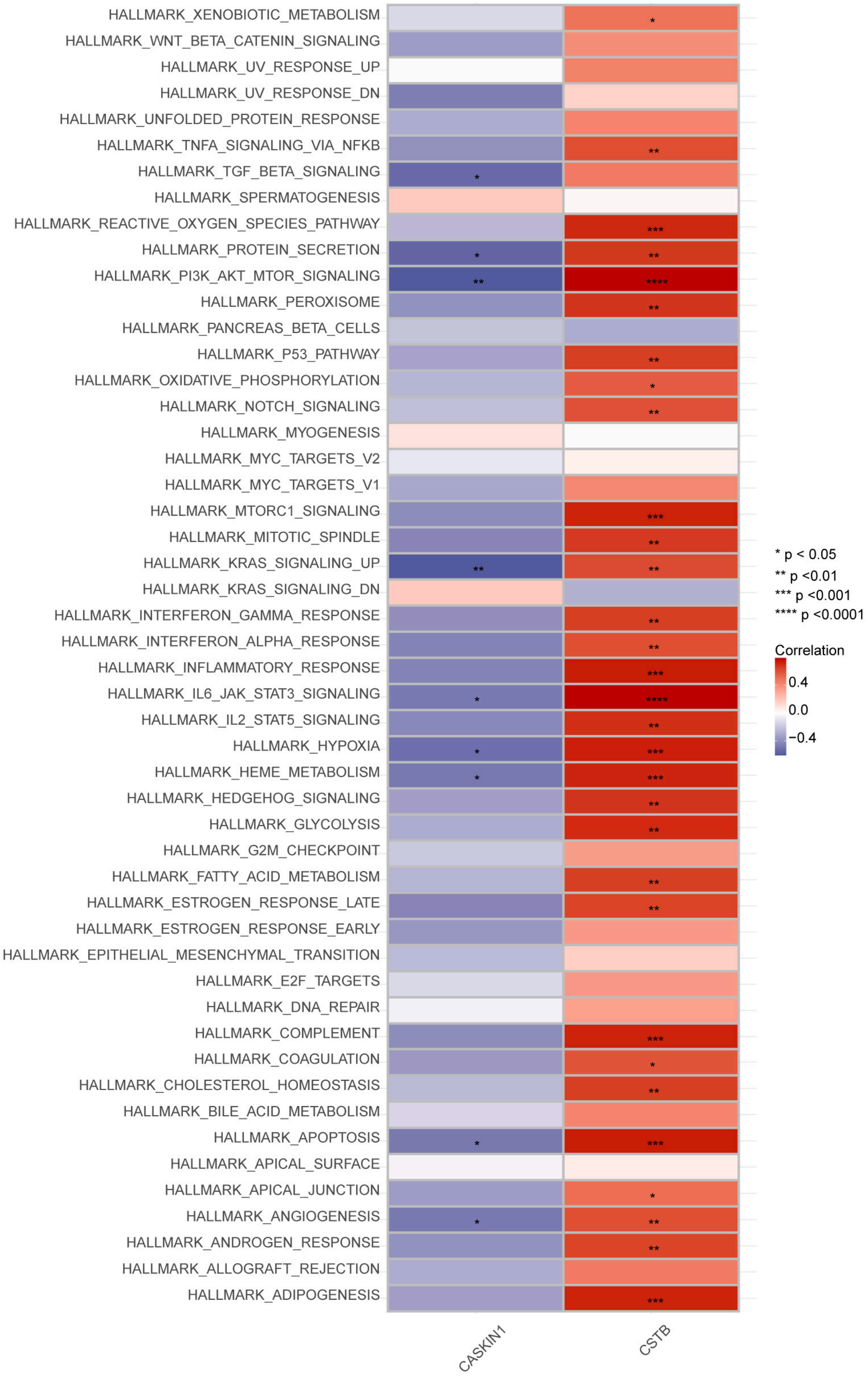

Figure S11. The correlations between 2 hub genes and the 50 HALLMARK signal pathways. ****$P < 0.0001$, ***$P < 0.001$, **$P < 0.01$, *$P < 0.05$.

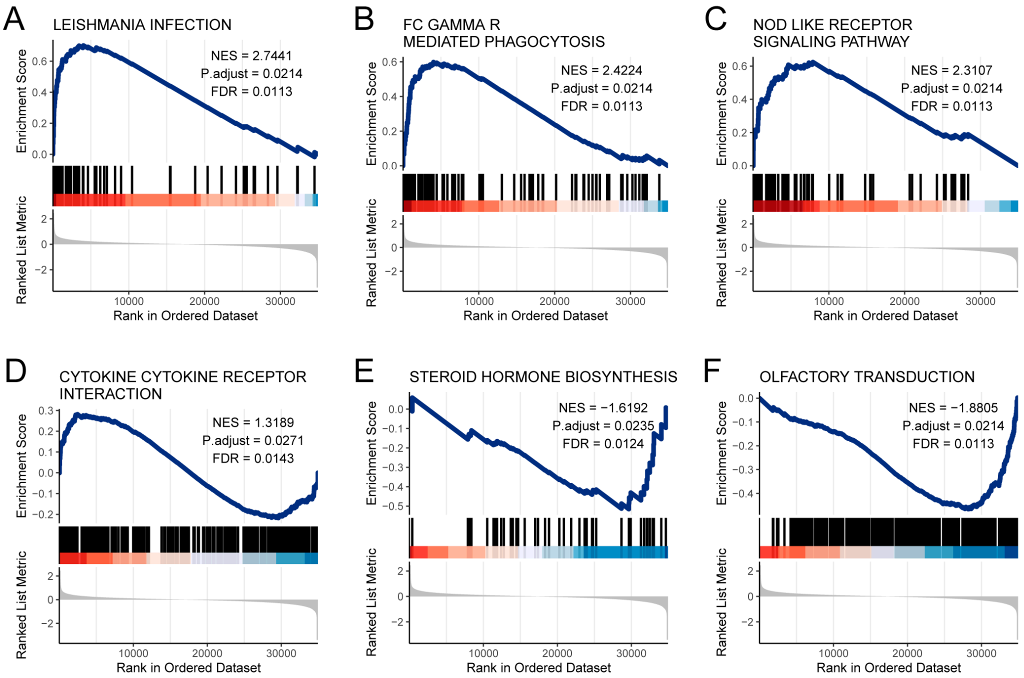

Figure S12. GSEA enrichment analysis of the DEGs. GSEA analysis revealed significant enrichment of (A) LEISHMANIA INFECTION, (B) FC GAMMA R MEDIATED PHAGOCYTOSIS, (C) NOD LIKE RECEPTOR SIGNALING PATHWAY, (D) CYTOKINE CYTOKINE RECEPTOR INTERACTION, (E) STEROID HORMONE BIOSYNTHESIS, and (F) OLFACTORY TRANSDUCTION. NES: Normalized Enrichment Score. GSEA: Gene Set Enrichment Analysis. The criteria for significant enrichment and screening in GSEA analysis were $P < 0.05$ and q-value < 0.05, respectively.
